# Misconceptions and paradoxes in soil-transmitted helminthiases control as a public health problem

**DOI:** 10.1371/journal.pntd.0006672

**Published:** 2018-09-13

**Authors:** Alejandro J. Krolewiecki

**Affiliations:** Instituto de Investigaciones en Enfermedades Tropicales, Universidad Nacional de Salta/Consejo Nacional de Investigaciones Científicas y Técnicas, Oran, Argentina; George Washington University School of Medicine and Health Sciences, UNITED STATES

## Abstract

Soil-transmitted helminthiases (STHs) constitute a public health problem that requires immediate action to resolve the morbidity of those harboring the parasites in their guts, to prevent infection in all those at risk, and to interrupt the vicious circle of poverty and disease in the affected communities, structural poverty being the main determinant of this group of infectious diseases. Since the times of the Rockefeller initiatives over a hundred years ago, the strategy has been viewed as one requiring community-wide efforts rather than pure individual case management. The World Health Organization (WHO) and its regional offices, as the governing institutions endorsed by the countries and their governments, have been the leaders in stating the actual executive measures to reach the goals and endpoints for the management of the problem. With the task of setting a group of activities that could be launched, monitored, and measured, these actions were established with the available resources since this public health problem had to be launched immediately, resources were those available at the moment and not those appearing on a wish list. Considerable progress has been made in the establishment of policies for the achievement of the Millennium Development Goals (MDGs), later followed by the Sustainable Development Goals (SDGs) through WHO-lead actions for the control of neglected tropical diseases (NTDs). With an initial goal of morbidity control, there are already discussions and proposals for elimination of STH if support is sustained and empiric facts confirm data emerging from modeling and small-scale studies. The aim of these comments is to describe and question instances of currently accepted concepts, theories, and practices that conform to the dogmatic status quo that serves as the foundation on top of which the new elimination aspirations are supposed to be built on, which might not be serving the desired purpose if taken unrevised.

## The sacred graph

Proper understanding of the principles guiding transmission dynamics of infectious diseases has been fundamental for disease control. Theories and models developed by Manson for filarial worms, Ross–Macdonald for malaria, and Anderson, May, and Schad for intestinal helminths are the foundation of currently more evolved models and have been confirmed by experimental data [1,2]. As described by Nobel laureate Daniel Kahneman, “…big breakthroughs in our mechanisms of understanding of association was an improvement in a method of measurement” [3]; this can be applied to our understanding of how worms perpetuate within their hosts and in the environment. Improvements in egg-counting methods in stools served to determine intestinal adult worm populations and the burden of parasites poured into the environment [1]. These concepts allowed the construction of a graph that still serves as the argument to establish the need for egg-counting methods for proper monitoring and follow up of interventions to identify significant changes in mean worm burden before prevalence starts to fall. A careful re-examination of the graph might not support the conclusions that are often accepted.

**Fig 1:**
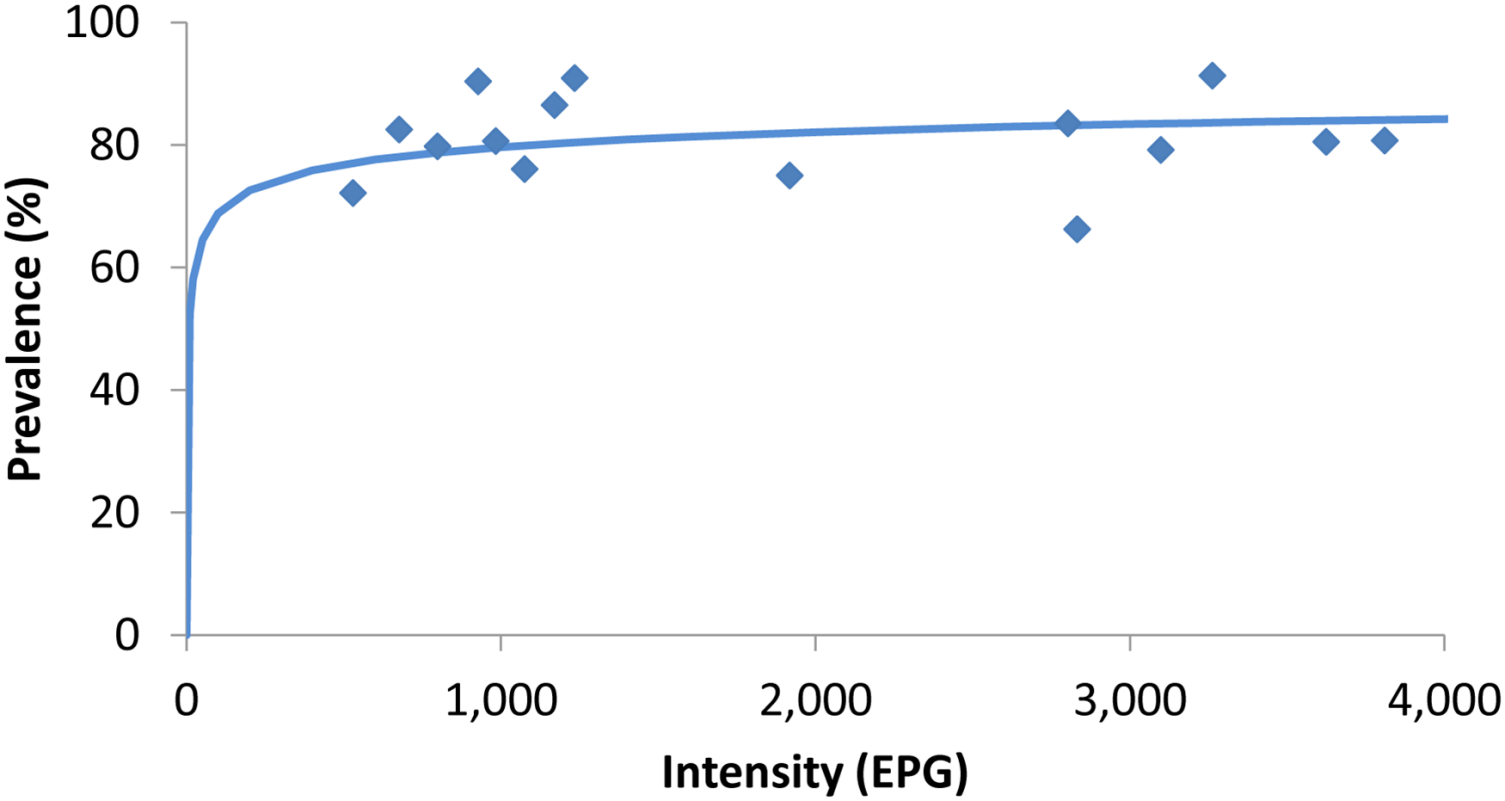
Source [5]. Binomial distribution curve describing the relationship between prevalence and intensity of infection in a study of *Ascaris lumbricoides*.

This curve (Fig 1), originally described by Guyatt and colleagues [4], best fitting a negative binomial distribution, is used as a dynamic expression of the evolution in the relationship between prevalence and mean worm burden. The assumption that would be incorrectly derived from this graph is that the response to an intervention would follow this curve since the graph was constructed not on temporal trends of any given community but rather on baseline surveys from varied geographies [4]. Whether the frequency distribution in response to an intervention reproduces this curve or not is still to be confirmed; changes in the speed of change and shape of the curves will likely differ with different efficiency of the drug regimen used, levels of water and sanitation, and migration dynamics, as examples of variables that could determine the impact of an intervention.

Another interesting aspect of this figure lays on the absolute prevalence values. With the curve acquiring a rather vertical shape at prevalence levels of around 70%, this value leaves most affected communities outside the “horizontal” part of the curve. Based on data from the Global Atlas for Helminth Infections, a query on baseline preintervention surveys from 2011 through 2015 revealed that, of 74 surveys, just a single survey for *Ascaris lumbricoides*, 2 for hookworms, and none for *Trichuris trichiura* had baseline prevalence >70% (Global Atlas of Helminth Infections [GAHI], query of preintervention surveys, 5 June 2017). In a review on preschool age children (PSAC), baseline prevalence >70% was determined for *A*. *lumbricoides* in 21% of 19 surveys, for *T*. *trichiura* in 10.5% of 19 surveys, and for hookworms in none of 15 surveys [6].

Given the arguments, it might be contended that qualitative diagnostic methods to estimate prevalence should provide all necessary information, saving the quantitative methods for studies involving what in Kahneman’s quote fulfills the concept of “mechanism”-like drug efficacy trials, resistance, and transmission dynamics studies. In a Markov model lead by WHO, it was demonstrated that proper estimations of the proportion of moderate- and high-intensity infections can be inferred from prevalence data [7]; these results could be interpreted as an indicator of the need of WHO to address a limitation in convincing field laboratory workers to obtain and register egg burdens.

## Underestimations in hookworm morbidity: The burden of low-intensity infections

The Global Burden of Disease (GBD) study ranks soil-transmitted helminthiases (STH) as the neglected tropical disease (NTD) with the greatest burden of years lived with disability (YLD), and among STH, hookworm stands out with the highest number of YLD (1.76 million) [8]. It is common sense to accept that, as more adult hookworms are attached to the intestinal mucosa, blood loss will be worse at any given baseline hemoglobin. Based on this, morbidity control is aimed at moderate- and high-intensity infections, obviating that these parasites produce chronic infections in populations living in environments with constant infectious events.

Two independent issues act to make proper morbidity measurements imprecise: coexisting endemicities that have anemia as a salient morbidity characteristic, like malaria, malnutrition, HIV/AIDS, and schistosomiasis, and second, the poor sensitivity of Kato–Katz in low-intensity infections [9]. These issues cause the misclassification of a substantial proportion of individuals as “noninfected” (false negative) in epidemiologic studies; therefore, the anemia that is attributable, at least in part, to hookworm disease is linked to the other comorbidities. Beyond anemia, more accurate diagnostic methods will probably uncover other aspects of hookworm-related morbidity, as recently demonstrated through Real-time PCR in the detrimental effect of hookworm coinfection among HIV-infected individuals in terms of CD4+ T-cell counts [10]. In Argentina, using multiple laboratory methods that improved overall sensitivity, anemia was a significant event in those harboring light hookworm (mostly *Ancylostoma duodenale*) infections. Moreover, relevant in reassessing the current policy despite having very few moderate- and high-burden infections (as is the rule across surveys from different geographies), significant impact in the prevalence of anemia was observed in all age groups after a community-wide intervention [11]. Improvements in diagnostic sensitivity (mainly in low-burden infections) might also uncover unrecognized morbidity due to *A*. *lumbricoides* and *T*. *trichiura*.

Finally, back to the GBD study, a subcategory of hookworm disease in the report lists “asymptomatic hookworm disease” with a global prevalence of over 350 million cases but zero YLDs; improved understanding of this condition might introduce further unrecognized morbidity [8].

## The single-dose myopia

Preventive chemotherapy (PC) through mass drug administration (MDA) is the mainstay and widely accepted strategy for STH control [12]. This is further described as a strategy executed through single-dose orally administered drugs. Taking the definition in pieces, it comes into question the mandate for single dosing. Unquestionably ideal, the least number of doses the better, with the aggregated benefit that single dosing allows witnessing the intake and reliable coverage indicators. A misconception around single-dosing arises when drug efficacy is ignored, and we are left accepting the current poor efficacies, which are unacceptable in the veterinary world, as good enough for our fellow humans [13]. Might we be underestimating their opinion on what they would rather take?

A review of mostly small studies revealed that albendazole increases its efficacy when used at repeated doses for hookworms and *T*. *trichiura* [14]. More recent trials have shown that albendazole at either 1 through 3 doses has cure rates against *T*. *trichiura* of 83% and against hookworm of 93% in the 3-day group, significantly different than 40% and 54%, respectively, with single doses [15]. Another study using 3-day regimens against hookworms showed significant improvements in the cure rate of albendazole for 3 days (45%) compared to a single dose (79%) (p<0.001) [16]. Steinmann and colleagues also demonstrated that 3-day regimens of mebendazole against *T*. *trichiura* achieved cure rates of 71% [17]. When taking a wider look at drug interventions for the control of infectious diseases in resource-limited settings, the same individuals not trusted to understand the importance and to effectively take the extra doses of anthelmintics following the dose that is witnessed by the health personnel is instructed to take, for a variety of periods up to a lifetime, drugs that, in most cases, have more severe acute side effects than benzimidazoles (Table 1). Might we be underestimating the capacity of the affected communities to understand what is best for them?

**Table 1:** 

Treatment durations
STH: single dose
Malaria: 3 days
Childhood pneumonia: 3 to 5 days
Tuberculosis: 6 months
HIV: lifetime

[12,18–20]

**Abbreviations**: STH, soil-transmitted helminthiases.

The strategy for the control of the HIV epidemic has set ambitious goals denominated 90-90-90, which means diagnosing 90% of infections, treating 90% of them, and achieving viral suppression in 90% of those treated [21]. Among the many coincidences with the STH control strategy, this initiative envisions elimination goals, includes rural communities, and relies on the key role of community healthcare workers [22]. However, while HIV/AIDS relies on open-ended daily treatment, STH aims for single-dose antihelminthics.

While perceived as more demanding to the programs, the higher efficacy of multiple-day regimens might abbreviate the number of MDA rounds and tablets needed to reach transmission interruptions.

## The minimum necessary acting as a glass ceiling

Updated WHO guidelines of PC for the control of STH extend the recommendation to PSAC, nonpregnant women of reproductive age (WRA), and pregnant women after the first trimester. The addition of these groups besides school age children (SAC), despite the a priori restricted use of donated drugs to SAC, sends the right message to public health authorities and program managers about goals, targets, and direction. Although it might seem contradictory in terms of not being able to match the resources offered with the recommendations issued, it sets a policy not tied to drug donations and offers guidance to governments and other stakeholders by addressing recommendations that were transforming a minimum set of activities and guidelines into a paradoxical glass ceiling to more ambitious strategies and initiatives.

## Coverage: Paradoxically not defined and closing remarks

Guidelines establish coverage as the outcome measure, without prevalence, morbidity, or other outcomes that constitute the ultimate goals of the interventions. A clear definition of this simple calculation, missing from all practice guidelines and glossaries, should allow stakeholders to know the proper denominator, help evaluations, and also determine how close programs are to the coverage rates used in modeling studies to predict the likelihood of transmission interruption [5,12].

The interconnected nature of the different issues outlined in this viewpoint state just some aspects that deserve re-evaluation. Dogmatic approaches are exposed with the aim of questioning the current status quo.
